# Putting rural health first in the fuel crisis

**DOI:** 10.1016/j.lanwpc.2026.101872

**Published:** 2026-04-30

**Authors:** The Lancet Regional Health – Western Pacific

The fuel shortage crisis rippling from the Middle East conflict since early March has exposed how vulnerable many countries are to energy disruption despite the distance from the war zone. In the Western Pacific region, economies such as Japan, Singapore, Taiwan, and South Korea almost fully rely on imported fossil fuels and more than 80% of the oil and liquefied natural gas transported via the Strait of Hormuz is destined for Asia. In the same month, the Philippines declared a national energy emergency, and Australia has drawn on its less-than-40-day national fuel reserve to dampen price surges in domestic markets. On April 8, 2026, a 2-week ceasefire was announced but no one can promise if it will hold given the century-long geopolitical tension. What is predictable are the impacts on civilians, which could include imminent health consequences on communities that are often overlooked.

A fuel crisis is not only a transport issue but also relevant to public health in the Western Pacific. The International Monetary Fund warned that a 10% increase in fuel price would boost inflation by 40 basis points and worsen today's cost-of-living pressure, and it may take weeks, if not months, for fuel prices to return to pre-war levels. In case of a sustained crisis, nutrition is likely to be hit the hardest as households struggle to afford fresh produce, healthy diets, and supplements and are forced to choose ultra-processed food when budgets tighten. The knock-on effects extend to physical activity and social health, as higher fuel costs have started to limit school excursions and outdoor experiences.

The threats affect everyone but are often felt the deepest by vulnerable communities. In East Asia and Pacific, 850 million population reside in rural areas. In remote communities, long-distance commutes add to existing geographical barriers and put patients at greater risk of delayed diagnosis and treatment. It likewise disrupts health workers’ outreach to remote patients and home-based aged care for older adults. Remote health initiatives, such as mobile clinics, screening trucks, and on-site diagnostics, may face higher operating costs that undermine the sustainability of services, let alone ongoing education programmes and research activities in rural settings. In Pacific Island countries where medical resources are geographically dispersed, health-care systems and patients might encounter challenges with cold-chain transport for vaccines and medications, as well as increased costs of both inter-island and international cross-boundary care.

Rural residents are known to have poorer mortality outcomes than their metropolitan counterparts globally. A meta-analysis shows that patients with cancer in rural areas have a 10% lower cancer-specific survival and a 15% lower all-cause survival, with disparities widening as geographical remoteness increases. In Aotearoa New Zealand, inequities in mortality rates between Māori and non-Māori populations are found to be amplified by the rurality of residence. Although recent initiatives inspired by the COVID-19 pandemic, such as telehealth solutions and mobile health stations, help to partly alleviate challenges with access to care, not all patients benefit as many still require in-person care. For example, rural patients with kidney failure often travel long hours to designated dialysis facilities several times a week; patients with rare diseases are referred to specialists at the quaternary level of care that are only available in limited locations, and sometimes national travel is required. Each macroeconomic shock is accompanied by a certain extent of health consequences, but the harm tends to distribute unevenly as the marginal effect of skipping one medical appointment among the neediest could be greater than skipping one meal in the cost-of-living crisis.

The fuel shortage crisis risks further widening urban–rural health inequities. The region should always prepare for the worst scenarios in case of persistent inflationary pain and risk of breaching a ceasefire by pre-emptively securing transport resources that prioritise frontline health workers and emergency medical services to ensure continuity of essential care. As a short-term measure, patients in rural settings who need to commute should be offered subsidised travel or accommodation assistance to ease their care-seeking process. In the longer run, governments should discuss alternatives and develop crisis management contingencies to strengthen system resilience and preparedness. The current model of care that blends digital and mobile health has addressed pressing needs in rural settings, but its resilience has been put to the test when resources to support mobility are no longer an option.

Countries in the Western Pacific may play a relatively passive role over the geopolitics in the far West, but at least we retain full control over how to use the resources on hand. Decisions made now on patient support, system resilience, and how we put the most vulnerable first could determine whether an energy shock turns into a lasting domestic health equity crisis.

